# Union Meeting of the Connecticut Valley Dental Society, the New England Dental Society, and the Connecticut State Dental Association, at Springfield, Mass., October 23, 24, and 25, 1889

**Published:** 1890-09

**Authors:** 


### UNION MEETING OF THE CONNECTICUT VALLEY DENTAL SOCIETY, THE NEW ENGLAND DENTAL SOCIETY, AND THE CONNECTICUT STATE DENTAL ASSOCIATION, AT SPRINGFIELD, MASS., OCTOBER 23, 24, AND 25, 1889.[1]

(Continued from page 491.)

EVENING session called to order at eight o'clock. President Brackett in the chair.

The discussion of Dr. J. A. Bazin's paper on "Limitations in Treatment and Filling of Roots" (for paper see page 522) was then opened by—

*Dr. Maxfield.*—As I have read over Dr. Bazin's paper and examined the specimens sent with it, I can perhaps speak more understandingly than those who have not yet had this opportunity. I think Dr. Bazin cannot have had much experience with the "Gates-Glidden" or "Morey" drills, or of "peroxide of hydrogen," or else he would not have seemed to have met so many difficulties. In the first place some of the teeth here presented have very tortuous roots: now no one would ever attempt to claim that the canals in such roots can be thoroughly cleaned. I do not doubt but what most of the failures that we do here in root-treatment are the result of this tortuous condition. Some of these specimens show a bend almost at right angles just below the apex of the root. If we thoroughly understand how to use the peroxide we will not have much trouble with these cases, for it will penetrate everywhere; and then, if we are unable to get all the *débris* out of the canals, we can so thoroughly saturate with an antiseptic that there is not likely to be any trouble from it. As to drilling out the canals, I always do that, using the drills mentioned. For molars I use the right-angle attachment, and have had some drills made for me a quarter of an inch shorter than those found at the depots. While drilling out the canals I keep the parts saturated with the peroxide, constantly withdrawing the drill to keep it clean. I am not, therefore, troubled with the dust remaining in the canals, as the writer seems to be. The weakness of the walls of the cavity I regard as of secondary consideration. The first question always is, "Is it best to attempt to save the tooth?" In ninety-nine cases out of a hun-

---

[1] Reported for the INTERNATIONAL DENTAL JOURNAL by Geo. A. Maxfield, D.D.S., Holyoke, Mass.

dred this will be answered affirmatively. Then we must sacrifice any part of the tooth that prevents our gaining access to the canals. I never attempt to treat a canal by working around a corner, but always cut away so as to gain free access. In filling the cavity we can strengthen the walls by using cement, filling it more than two-thirds with this material, letting it run down the walls almost to the edges of the cavity, then covering either with amalgam or gold. In all large cavities, whether or not in pulpless teeth, I think this much the best way to fill. Only in case of abscess is it necessary to drill through the end of the root. If we have been careful in cleaning out the canal there will be no *débris* to be forced through with the drill. In all cases I prefer that the peroxide shall penetrate the canal ahead of my instrument, and I use it very freely. By using these precautions and with care I do not think we will find many roots of teeth whose formation will so limit our treatment that we shall be unable to save the tooth.

Discussion closed.

(To be continued.)

